# Supplementary material for: Sequential Membrane Filtration to Recover Polyphenols and Organic Acids from Red Wine Lees: The Antioxidant Properties of the Spray-Dried Concentrate
Source: Membranes (Basel). 2022 Mar 23;12(4):353. doi: 10.3390/membranes12040353 (PMC9030477; doi:10.3390/membranes12040353)
Supplement: Supplementary file 1 [file membranes-12-00353-s001.zip › membranes-1608748-supplementary.pdf]

membranes

Sequential Membrane Filtration to Recover Polyphenols and Organic Acids from Red Wine Lees: The Antioxidant Properties of the Spray-Dried Concentrate

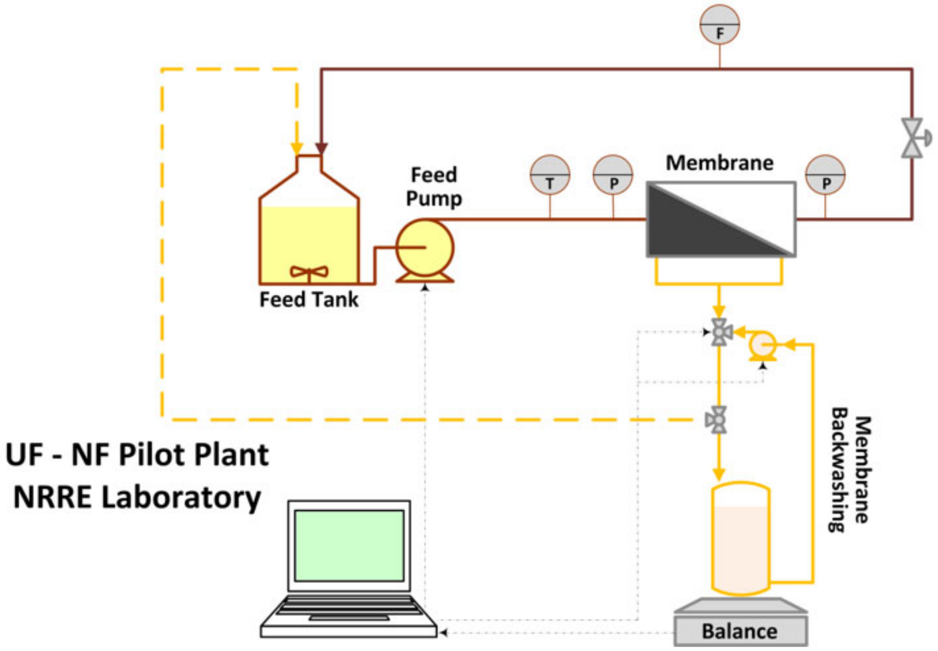

Figure S1. Custom made laboratory-scale cross-flow filtration pilot unit.

==== Shimadzu LCsolution Calibration Curve ====

<Calibration Curve>

ID#	: 1
Name	: L-tartaric acid
Quantitative Method	: External Standard
Function	: f(x)=1211.12*x+2093.76
	Rr1=0.9990222 Rr2=0.9980453
	MeanRF:1233.35 RFSD:85.9492 RFRSD:6.96876
FitType	: Linear
ZeroThrough	: Not Through
WeightedRegression	: None
Detector Name	: PDA

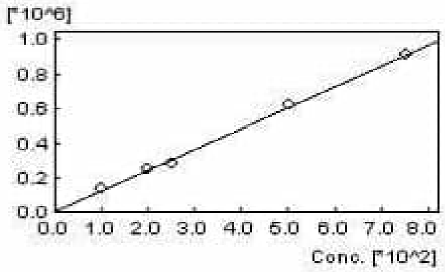

#	Conc (Ratio)	Area	Area
1	101.000	136147.4	136147
2	202.000	247132.9	247133
3	252.000	254400.5	254401
4	501.000	617999.1	617999
5	752.000	912492.5	912492

ID#	: 2
Name	: Dl-malic acid
Quantitative Method	: External Standard
Function	: f(x)=614.951*x-5514.09
	Rr1=0.9994785 Rr2=0.9989572
	MeanRF:592.943 RFSD:22.9377 RFRSD:3.86846
FitType	: Linear
ZeroThrough	: Not Through
WeightedRegression	: None
Detector Name	: PDA

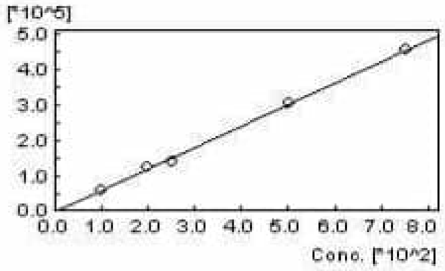

#	Conc (Ratio)	Area	Area
1	101.000	58695.1	58695
2	202.000	123027.2	123027
3	252.000	140583.5	140584
4	501.000	305332.4	305332
5	752.000	456622.5	456622

Figure S2. Calibration curves for tartaric and malic acid.

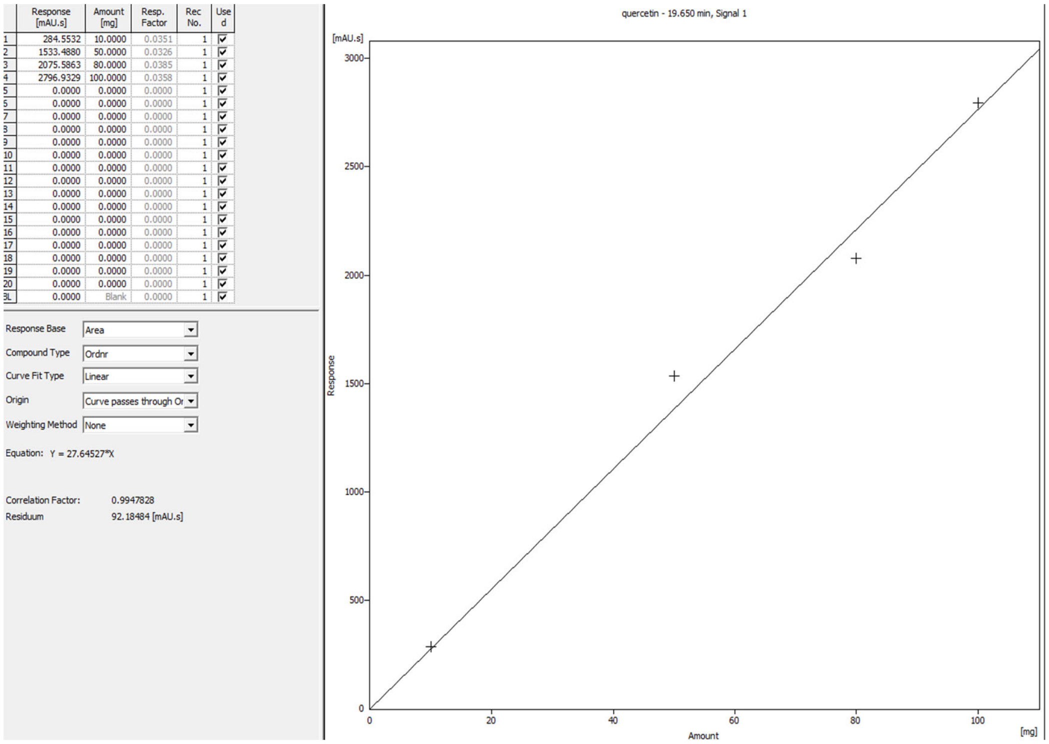

Figure S3. Calibration curve of quercetin.
